# Music intervention in precision shooting: mechanisms, effects, and future directions—a literature review

**DOI:** 10.3389/fspor.2025.1711610

**Published:** 2025-12-08

**Authors:** Yuqian Lan, Yubo Wang, Wenqiang Wu

**Affiliations:** School of Education, Beijing Sport University, BeiJing, China

**Keywords:** music, shooting performance, shooting, elite shooter, arousal

## Abstract

Previous studies have consistently shown that non-invasive musical stimulation can facilitate physical performance, particularly through fast-tempo, high-volume, and rhythmically strong music. Yet, it remains unclear whether slow-tempo, moderate-volume music exerts only inhibitory effects, or whether it might offer distinct benefits in certain contexts. In precision sports such as shooting, athletes must maintain dynamic balance of the body and firearm, physiological and psychological stability, and fine neural regulation. Whether fast-tempo, high-volume stimulation disrupts this optimal state is still unknown. This study therefore examines the bidirectional effects of non-invasive musical stimulation on shooting performance, with a focus on underlying mechanisms, potential benefits, and future directions, and specifically investigates whether slow-tempo, moderate-volume music may more effectively enhance shooters' performance compared to fast-tempo, high-volume stimulation.

## 1 Introduction

Non-invasive musical stimulation has been intertwined with human physical activities for millennia, from ancient ritual chants to martial drumbeats. In modern sport, music is nearly ubiquitous: stadiums blare pump-up songs, and many athletes train or warm up with headphones on. Scientific interest in how music affects athletic performance dates back over a century. As early as 1911, an American researcher, Leonard Ayres, observed that cyclists pedaled faster when a band was playing music than when they were in silence (1). Since then, dozens of studies have explored the interplay of music and exercise, revealing that music can distract from fatigue, elevate mood, enhance endurance, reduce perceived exertion, and even improve metabolism efficiency (2). In a 2012 review, Karageorghis famously described music as “*a type of legal performance-enhancing drug”* for athletes. In short, the right tunes can measurably boost many aspects of physical performance.

However, music's ergogenic benefits are not universal; they depend on how it is applied and on the nature of the sport or task. Researchers have found that *music's positive effects are most evident in self-paced, rhythmic, or endurance activities*, such as distance running (3) or cycling (4). Upbeat, synchronous music can improve work output and delay fatigue during repetitive exercise by narrowing attention and prompting movement synchronization (5, 6). Listening to motivating music also increases arousal and neural activity, which correlates with improved speed or strength in high-intensity efforts (2, 7). When exercise intensity reaches the maximal oxygen uptake threshold or anaerobic threshold, the mind becomes preoccupied with physiological strain, and music no longer mitigates perceived exertion (5, 8). Likewise, not all studies have found benefits—some report little to no improvement—highlighting that factors like music selection and personal preference are critical (6, 9, 10). Indeed, if the music is unappealing or inappropriate for the context, it may fail to help or even become a distraction (11). It is undeniable that music brings numerous interesting and healthy benefits to our lives. For instance, it can enhance athletic performance, as previously discussed. Furthermore, scholars have further proposed that music can effectively facilitate the implementation and execution of sophisticated tasks by surgical teams in the medical field. Meanwhile, classical music played at a low to moderate volume can significantly improve both the accuracy and speed of task completion. This effect aligns with the psychological and physiological demands required for shooters to successfully accomplish their shooting tasks (12).

An important moderating factor is the *type of athletic task*, especially its skill demands and required arousal level. In activities requiring gross motor skills and high power output (sprints, heavy resistance exercise, etc.), higher arousal from fast-paced music can be beneficial (13, 14). In contrast, sports that involve fine motor control, precision, and calm concentration often suffer if arousal is too high (14). Competitive shooting is a prime example of the latter. Olympic-style shooting (e.g., air rifle and pistol events) is characterized by low-intensity physical effort but extreme demands on steadiness and accuracy. Athletes must control their breathing, heart rate, and even emotions to keep the gun as still as possible and execute each shot with minimal error. Heart rate in elite shooters is typically low and stable during aiming, because even a slight pulse-induced tremor can throw off a shot (15–17). Mental focus is paramount: shooters train to maintain *calm, focused attention* for extended periods and to avoid distractions or anxiety that could disturb their fine motor control. In competition, they often face long qualification rounds (e.g., 60 shots in 75 min) followed by high-pressure finals, which test not only physical endurance (in maintaining posture) but also “neural endurance” to sustain concentration under stress. Intense emotions—whether anxiety, excitement, or frustration—can be detrimental, as they trigger physiological responses (e.g., racing heart, muscle tension) that degrade aim stability and decision-making (18, 19). Thus, optimal performance in shooting is achieved in a state of *low arousal but high focus*: a controlled psychological zone sometimes described as being “calm yet alert”.

Given these unique demands, the idea of using music to aid performance in shooting might seem counter-intuitive at first. Many athletes use music to energize and psych-up, but a shooter generally does not want to be *too energized*. The wrong music (for instance, a fast, adrenaline-pumping song) could break a shooter's carefully cultivated calm, causing heart rate fluctuations, jitteriness, or distraction at the worst possible moment. On the other hand, the *right kind of music* might help a shooter in several ways: by reducing pre-competition anxiety, by elevating a depressed mood, by blocking out distracting noise or thoughts, and by facilitating a smooth pre-shot routine (e.g., via imagery or rhythmical breathing). In recent years, athletes and coaches in precision sports have indeed begun experimenting with pre-competition music as a tool to get “in the zone”. For example, some archers and shooters listen to calming background music before or between shots to maintain steady nerves. Early evidence specific to precision sports is limited but suggestive. A pilot study with elite pistol shooters found that listening to relaxing music during mental practice (imagery) led to lower physiological arousal, whereas arousing music increased arousal levels, in line with the intended effects (14, 20). Another experiment, on netball shooting accuracy, reported that self-selected motivational music produced no significant improvement in shooting performance compared to no music, highlighting that in skilled aiming tasks the benefits of music are not automatic​ (20, 21). These mixed findings underscore that music intervention in sports like shooting must be carefully tailored.

This article provides a comprehensive review of how pre-competition music interventions might influence the performance of competitive shooting athletes. We examine the underlying mechanisms by which music can affect an athlete's physiology, psychology, and motor control, with an emphasis on the context of shooting. We then discuss practical intervention strategies—such as selecting appropriate music tempo, style, and timing—for integrating music into shooters' training and pre-shot routines. Next, we identify current research gaps and future directions for this emerging interdisciplinary topic, including the need for sport-specific studies and neurophysiological investigations. Finally, we offer a conclusion summarizing key insights and recommendations. Through this multidimensional analysis, we aim to deepen the theoretical understanding of music's role in sports psychology and provide actionable guidance for harnessing music to improve precision performance in competitive shooting.

## 2 Mechanisms: how music influences arousal, emotion, and performance

### 2.1 Physiological and neurological mechanisms

**Figure 1:**
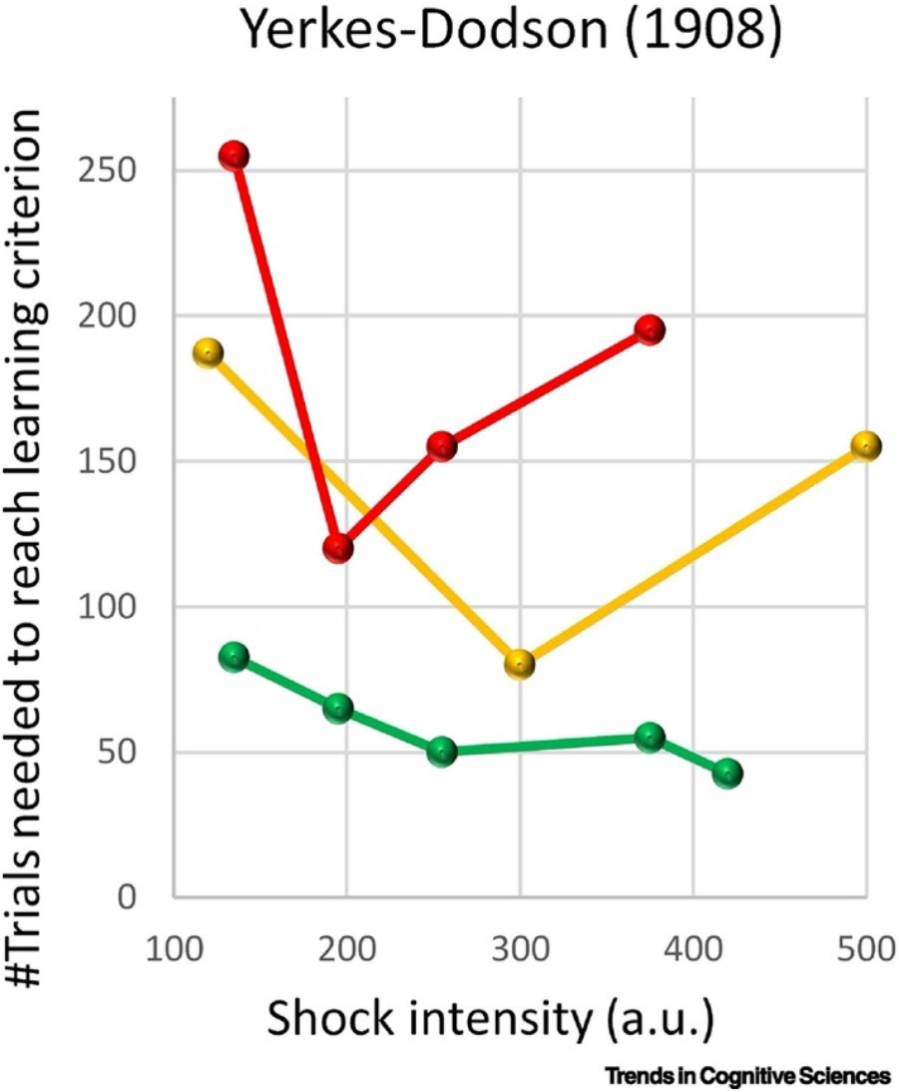
Original data reported by Yerkes and Dodson(27). Horizontal axis (*x*-axis): Shock intensity, in arbitrary units (a.u.).Vertical axis (*y*-axis): Number of trials needed to reach the learning criterion (i.e., fewer trials = faster habit formation). Green line: the number of trials falls nearly monotonically as shock intensity increases—thus stronger stimulus → faster learning, up to the highest intensities shown. Yellow line: the curve shows a moderate decline in trials with increasing intensity, but the effect levels off or slightly reverses at very high intensities. Red line: the curve exhibits a U-shaped pattern—at low stimulus, trials required are high (learning slow); as stimulus increases to a moderate level, required trials drop (learning faster); beyond that moderate level, required trials increase again (learning slows).

Music has direct effects on the human nervous system and physiology, which in turn can impact athletic performance (22). When one listens to music, auditory patterns are processed in the brain's auditory cortex and limbic system, triggering changes in autonomic nervous activity and hormone secretion (23). Up-tempo, vigorous music (especially with a strong beat) tends to stimulate the sympathetic nervous system—the “fight or flight” branch—leading to increases in heart rate, blood pressure, and adrenaline (epinephrine/norepinephrine) levels (24). This response can be beneficial for athletes about to engage in explosive or high-intensity activities, as it primes the body for action. However, in a shooter who needs to remain calm and still, excessive sympathetic arousal is counterproductive (25). An elevated heart rate can impair fine motor steadiness by exacerbating the slight body movements with each heartbeat (15). High adrenaline and muscle tension can undermine the smooth trigger squeeze and breath control that marksmanship requires. In other words, high arousal creates physiological “noise” that is detrimental to precision (14, 19). Indeed, sport science recognizes that tasks involving fine motor control usually see performance deteriorate under conditions of over-arousal (14, 26). This aligns with the classic Yerkes-Dodson “inverted-U” principle, like Figure 1—shooting lies on the low-arousal end of the curve where a calm state yields optimal results.

**Figure 2:**
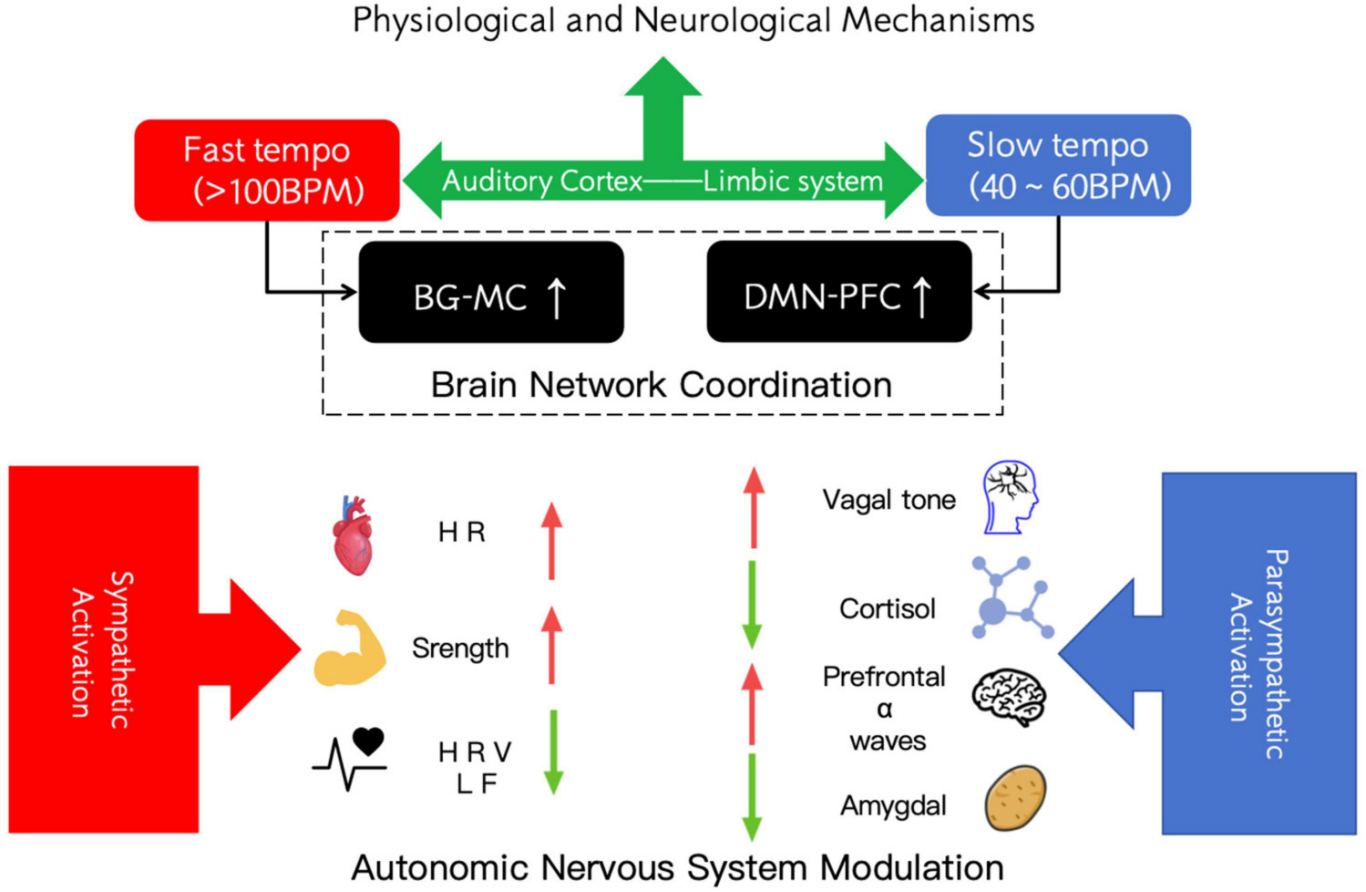
Physiological and neurological mechanisms. BG-MC, basal ganglia—motor cortex network; DMN-PFC, default mode network—prefrontal cortex network; HR, heart rate; HRV LF, heart rate variability—low frequency component; Vagal tone: vagal toneparasympathetic vagus nerve activation; prefrontal α waves, prefrontal alpha waves.

Conversely, slower, sedative music can activate the parasympathetic system—the “rest and digest” response—which helps bring the body to a calmer physiological state. Research has shown that listening to slow, relaxing music after strenuous exercise accelerates the return of the body to baseline: it lowers heart rate more quickly and even tends to reduce cortisol (a stress hormone) levels compared to not listening to music or to fast music (26, 28, 29). In one experiment, slow music during post-exercise recovery led to significantly larger drops in arousal, faster heart-rate recovery, and more positive emotions, whereas fast music actually *slowed* the reduction of heart rate and decreased blood lactic acid (30). Extrapolating these findings to a pre-performance setting, one can infer that calming music helps create a low-stress physiological profile: lower circulating stress hormones, a more stable heart rhythm, and a general state of relaxation. This is exactly the kind of internal environment a shooter wants going into competition. Furthermore, music's rhythm can interact with the athlete's own biological rhythms. There is evidence that people tend to unconsciously synchronize aspects of their physiology (breathing rate, for example) with musical beats (31–33). If the music's tempo is appropriately matched, it may encourage slower, deeper breathing and a lower heart rate, reinforcing the relaxation response. On the neural level, relaxing music has been associated with increases in low-frequency brain waves (such as alpha or delta waves) indicative of calm, meditative states, and with decreased activation of brain regions linked to fear and anxiety (such as the amygdala) (14, 34, 35). As summarized in Figure 2, music can act as a tool to tune the shooter's physiological state, dialing it down to an optimal “comfort zone” in which the body is primed for steady aiming rather than explosive movement.

### 2.2 Psychological mechanisms

In addition to its bodily effects, music profoundly influences the psychological state—emotions, arousal level, attention, and motivation—all of which can affect sports performance. Athletes across disciplines use music strategically to manipulate their mood and mindset before competitions (14, 36). For example, an athlete feeling sluggish might play an upbeat, inspiring song to increase alertness and motivation, whereas one who feels anxious might choose a soothing piece to foster calmness. These psychological effects are highly relevant in shooting. Competitive shooters often struggle with performance anxiety and overthinking, especially in high stakes moments like a final or a tie-breaking “shoot-off”. Here, music can serve as a form of emotion regulation. Listening to preferred calming music prior to shooting has been reported to lower subjective anxiety and nervousness, helping athletes attain a more optimal emotional state (low anxiety, positive mood) (37). Research in sports psychology shows that music can indeed help narrow an athlete's focus of attention, essentially shielding the mind from intrusive worries or external distractions (38). In shooting, where concentration and attentional control are paramount, this could be a valuable benefit: the music provides a benign stimulus for the mind to latch onto, preventing it from ruminating on fear of failure or the pressure of competition. However, this must be balanced carefully—the music should not be so engaging as to divert the shooter's attention away from task-relevant focus. That is why usually music is used before or between shooting series, not during the aiming of a shot. The goal is to achieve the right psychological *zone* at the start of the performance.

Another psychological mechanism of music is its impact on imagery and cognitive preparation. Mental imagery (or visualization) is a common technique in shooting training: athletes vividly imagine themselves performing perfect shots, which enhances neural preparedness and confidence. Studies indicate that adding music to imagery can enhance the experience. For instance, there is evidence that relaxing background music can make imagery more effective by facilitating appropriate arousal levels and emotional engagement (14, 39, 40). Many guided imagery recordings for athletes deliberately include soft music to set a calming backdrop (41). In the context of shooting, a pre-competition routine might involve the shooter sitting quietly with noise-cancelling headphones, mentally rehearsing their shot process while listening to a gentle piece of music. This pairing can synergistically reinforce the shooter's *ideal performance state*: the music keeps them relaxed and focused, while the imagery activates the precise motor patterns and confidence they need for competition. A pilot study by Kuan et al. with elite shooters supports this approach: when shooters listened to soothing classical music during imagery, physiological indicators confirmed a reduction in arousal (lower skin conductance and muscle tension), without compromising the vividness of their mental rehearsal (14). Thus, music can enhance psychological skills practice and pre-shot routines, which in turn improves actual performance.

It is worth noting that the effects of music on emotion and motivation are highly individual. The emotional response to a given song can differ greatly from person to person based on their memories, cultural background, and personal associations with that music (4). A melody that calms one shooter might irritate or sadden another. For this reason, the psychological mechanism of *preference* is key: *self-selected music* (chosen by the athlete) almost always yields stronger positive effects on mood and performance than music imposed by someone else (42, 43). Athletes are more motivated and emotionally affected by music they love, whether that's a classical piece, a religious hymn, a hip-hop anthem, or a heavy metal track. What matters is that the music carries positive meaning and appropriate energy *for that individual*. This observation has important implications for designing interventions, as we will discuss in the next section.

In summary, music can influence shooting performance through multiple interconnected pathways. Biologically, it adjusts the autonomic balance, hormone levels, and brain activity to either pep up or calm down the athlete. Psychologically, it alters arousal, emotion, and focus, shaping the mental state with which the athlete approaches their task. The optimal scenario for a shooter is one where music induces a *psychophysiological state of calm concentration*: heart rate and breathing are steady, muscles are relaxed yet responsive, the mind is clear of anxiety, and confidence is high. Achieving this state can help the shooter execute each technical element (aiming, trigger control, timing) with greater consistency. On the other hand, a poorly matched music choice (too fast, too chaotic, or simply not to the athlete's liking) could overshoot arousal or become a distraction, thus impairing performance. The mechanisms above underline why music must be carefully tailored to the athlete and context. Next, we turn to practical considerations for implementing music interventions for competitive shooters to maximize the benefits and minimize any downsides.

## 3 Implementation of music interventions in shooting: strategies and considerations

Designing an effective pre-competition music intervention for a shooter requires translating the mechanistic insights into actionable strategies. Broadly, three dimensions should be considered: tempo, volume and personalization, These factors help ensure that the music aligns with the athlete's physiological needs and psychological preferences. Below, we outline each component and how it can be optimized for shooting sports.

### 3.1 Tempo

The tempo or rhythm of the music is a critical factor in how it affects arousal. Fast-tempo music (e.g., >120 beats per minute) tends to be stimulating, elevating heart rate and adrenaline, while slow-tempo music (≈60–90 bpm) tends to be relaxing, lowering heart rate and blood pressure (26, 44). For shooters, the *beat of the music should be compatible with the desired pre-competition heart rate and mood*. Typically, this means favoring slower, steady tempos that mimic a calm heartbeat. For example, an athlete whose resting heart rate is around 80 bpm might choose music in the 70–100 bpm range to foster a subtle entrainment—the body's rhythms synchronizing with the music's rhythm. Such entrainment can promote a stable physiological state; the shooter might subconsciously slow their breathing and heart rate to “match” the music. Research on rhythm response suggests that most people naturally synchronize movements (or even neural firing patterns) with musical beats (20). In practical terms, shooters should avoid very fast, frenetic songs right before performance, as these could push arousal beyond the optimal zone for fine motor control. Instead, moderately slow songs with a smooth, consistent beat are ideal for creating a relaxed yet alert state. It's also helpful if the rhythm can facilitate a pre-shot routine—for instance, a shooter might time their breathing or visualization exercises to the gentle cadence of the music. A stable beat gives a sense of structure and can mentally prepare the athlete for the rhythmic process of competition shots (which often follow a repeatable cycle of raise the gun—hold breath—trigger—follow-through). In short, tempo-tuning the music to the athlete's internal rhythms helps establish physiological comfort and focus.

### 3.2 Volume

The volume at which music is played can influence its effects. Very loud music (>90 dB) might induce excitement but can also raise stress and even cause discomfort or hearing damage, whereas very soft music might be drowned out by ambient noise and fail to engage the listener. For pre-event music in a controlled environment (like a waiting area or warm-up hall), a moderate volume (∼70–80 dB) is often recommended—loud enough to immerse the athlete in the music, but not so loud as to be overwhelming or to prevent the athlete from thinking quietly (20, 26). Using noise-canceling headphones can help create a personal “audio bubble”, minimizing outside interruptions. Many athletes already use headphones for their pre-game music; for shooters, headphones can double as a tool to block distracting chatter or sudden noises that might spike anxiety. It's important that the volume is comfortable because comfort aids relaxation. Some shooters prefer starting with slightly higher volume to block out everything else, then gradually lowering it as they get closer to the competition start, easing the transition to the relative quiet of the shooting range. Volume control also ties into safety and awareness—while preparing, the athlete may still need to hear announcements or cues from coaches/judges, so having music at a controllable volume (or one earbud out) could be practical. Fade-in and fade-out techniques are useful: a slow fade-out of music as the athlete steps to the line can leave them with a residual sense of calm from the music without an abrupt cutoff. In training sessions, athletes might also practice with music at different volumes to see what works best for maintaining focus. Ultimately, volume should be set such that the music is an aid, not a hindrance—it should *soothe and center* the shooter, not startle or saturate them.

### 3.3 Personalization

As noted, individual preference plays a huge role in the efficacy of music interventions. Therefore, constructing a *personalized music playlist* is essential. The athlete should select songs that they *enjoy, find motivating or calming (as needed), and that carry positive associations*. The emotional resonance of the music can amplify its effect—songs that uplift the athlete's mood or confidence will enhance the pre-competition mindset. Conversely, a song that the athlete dislikes or that evokes unwanted emotions could be detrimental. In practice, sports psychologists often work with athletes to develop a music library tailored to their taste and performance needs. One evidence-based tool for this is the Brunel Music Rating Inventory (BMRI), which evaluates the motivational qualities of songs (factors like rhythm, melody, tempo, style) to help identify suitable tracks (20, 45). While the BMRI has been used mostly for upbeat exercise music, the same principles apply to selecting *relaxing tracks*—the music should have an appropriate tempo (as above), simple and soothing melody, and lyrics (if any) that have a positive or at least neutral emotional effect. Notably, *lyrics in one's native language* can strongly draw attention; some shooters may prefer instrumental music or songs in a language they don't fully understand to prevent their mind from fixating on lyrical meaning. Other athletes might find certain lyrics empowering. These nuances underscore personalization. Another consideration is cultural relevance: an athlete from one culture might relax to the sounds of a traditional folk song or classical piece, whereas another might prefer contemporary soft rock. Studies have shown that cultural background influences rhythm preferences and the emotional impact of music (9, 46, 47). Thus, the music library should be built through experimentation—the athlete tries different songs during training to see which ones consistently produce the desired mental/physical state. Over time, listening to the same “favorite” pre-competition songs can become a conditioned cue for the body to relax and the mind to get into focus, a form of Pavlovian conditioning that further enhances consistency. In sum, *personalized, meaningful music* maximizes the psychological benefits (enjoyment, comfort, motivation) and avoids the pitfall of one-size-fits-all music prescriptions.

Beyond these three core factors, a few additional practical points merit mention. Timing of music use is flexible: some athletes listen continuously up until they take their first shot, while others stop the music a few minutes earlier to gather their thoughts in silence. This can be individualized; however, it's generally not allowed to listen to music during actual competition shots for fairness and safety reasons (in many events, shooters must wear ear protection that does not include music-playing capability). Therefore, the intervention is truly *pre-competition* or during breaks, and athletes should rehearse the process of removing headphones and carrying over the calm state into the silent competition arena. Also, coordination with warm-up routines is beneficial. For instance, shooters often do dry-firing (practicing trigger pulls) or light physical warm-ups before competing. Pairing these activities with appropriate background music can enhance their effectiveness—upbeat music during a physical warm-up to raise body temperature slightly, then switching to slow music during mental warm-up to lower anxiety. The transition from one track to another can itself be a cue (e.g., a shooter might always play a particular “theme song” as the final tune which signals them to enter full focus mode). Finally, coaches and sport psychologists should ensure that the athlete practices their music routine under various conditions so that on competition day it feels routine and not disruptive. When these strategies are applied, music becomes a powerful component of the shooter's pre-performance routine: it psychologically isolates them from the chaos of competition, puts them in their optimal arousal zone, and reinforces their well-practiced mental imagery and focus techniques, all of which can contribute to more stable and high-level performance.

## 4 Future directions for research and practice

While the existing evidence and strategies discussed are promising, the domain of music intervention in precision sports like shooting is still relatively nascent. Several avenues for future research and development emerge from the current review.

### 4.1 Sport-specific and individualized research

Many conclusions about music's effects on performance are drawn from general exercise studies or sports like cycling, running, and weightlifting. There is a need for more studies *directly involving shooting sports and other precision tasks*. For example, controlled experiments could examine the effects of different music conditions (tempo, genre, etc.) on actual shooting performance metrics (score, shot group consistency) in both training and competition settings. Such studies should also account for differences among shooting disciplines (rifle vs. pistol, air gun vs. firearm, etc.). The biomechanics and psychological stressors can vary between, say, rifle three-position shooting and rapid-fire pistol. It's plausible that music that helps in one discipline might not be ideal for another. Future research should parse out these nuances, perhaps developing discipline-specific guidelines. Moreover, individual differences in response to music warrant systematic study—for instance, measuring physiological responses (heart rate, HRV, cortisol) and performance outcomes in a group of shooters to identify *responders* vs. *non-responders* to music. This could help tailor interventions to those who benefit most, and develop profiles (based on personality or anxiety levels) for who is likely to find music useful as a pre-shot routine aid.

### 4.2 Long-term effects and neural adaptation

Almost all research so far has looked at immediate or short-term effects of music (acute interventions). An intriguing question is whether repeated use of music in training can lead to longer-term adaptations in the athlete's stress response or neural circuitry. Music engages reward pathways in the brain (releasing dopamine) and can induce neuroplasticity changes with consistent practice (48–50). Could regularly training with calming music improve a shooter's baseline ability to regulate arousal and emotion, even when music is not present? Perhaps the music-associated relaxation response becomes conditioned and easier to invoke over time. Studies using neuroimaging and EEG could explore how the brain's emotion and attention networks in shooters evolve after a season of incorporating music into mental training. If we find that certain neural markers (for example, heightened frontal alpha waves or reduced amygdala reactivity) are developed, it would indicate a lasting benefit of music-based interventions beyond the momentary performance boost. In addition, longitudinal studies could test whether integrating music into pre-competition routines over weeks or months yields performance improvements in competition compared to a control group that uses standard routines without music. This would address causality and the *training value* of music, not just its one-off effects.

### 4.3 Mechanistic insights via multimodal monitoring

To deepen our understanding of how exactly music exerts its influence, future work should employ *multimodal monitoring techniques* during music intervention experiments. This could include physiological sensors (heart rate monitors, HRV analysis, galvanic skin response for sweat, EEG for brainwaves, fNIRS for cortical blood flow, etc.) used simultaneously while an athlete is listening to music and preparing to perform. By correlating these objective measures with performance outcomes, researchers can build a more precise “*rhythm–emotion–neurology” model* as alluded to earlier. For example, we might discover that a certain range of heart rate variability induced by music is predictive of optimal shooting performance, or that specific EEG patterns (perhaps increased synchronization in frontal regions associated with focus) occur reliably when music successfully puts an athlete in the zone. With advanced signal processing and machine learning, it could even be possible to personalize music in real-time: biofeedback systems that adjust the music selection or tempo based on the athlete's current physiological state, steering them toward the desired state. Such interdisciplinary research, bridging sports science, psychology, neuroscience, and even musicology, would provide cutting-edge insights and more quantitative guidelines for practitioners.

### 4.4 Cultural and contextual factors

Another direction is exploring how cultural context and musical background influence the effectiveness of interventions. As mentioned, what is considered “relaxing” or “motivating” music can differ widely across cultures. Future studies might compare athletes from different countries or musical traditions to see if responses differ when using culturally familiar music vs. foreign music. Additionally, the *context* in which music is used could be studied: for instance, is music more helpful in training (when learning or refining skills) or in competition (when managing stress)? Does listening to music in a team setting (even in an individual sport like shooting, athletes often train in groups) have any social or cohesion benefits, or is it strictly an individual phenomenon? While our focus has been pre-competition music, some athletes use music *post-competition* to unwind and recover mentally—research could also consider if that indirectly benefits subsequent performance by improving overall well-being and recovery via reduced stress.

### 4.5 Integrating music with other psychological skills

Music intervention should not be viewed in isolation but as part of a toolkit of mental skills training. Future work could examine how music synergizes with or compares to techniques like deep breathing exercises, meditation, progressive muscle relaxation, or self-talk strategies in shooting. For example, does music combined with guided breathing yield better calming effects than breathing alone? Or could playing a background track during biofeedback training help athletes learn to control their physiology faster? Understanding these interactions will help coaches create comprehensive pre-performance routines. On the flip side, we must also explore any potential downsides—e.g., does reliance on music create a dependency such that an athlete might underperform if their music is unavailable? Ensuring that athletes can adapt if, say, a device fails or external music is banned is important. Future guidelines might recommend developing a “backup” mental routine without music for such scenarios.

In summary, the future of research on music in competitive shooting should be multidimensional, blending empirical performance trials with physiological monitoring and personalization. There is much to be discovered about the fine details of why and for whom music works in this context. The ultimate goal of these future directions is to refine our theoretical models and translate them into evidence-based practices that coaches and athletes can confidently use. With further study, pre-competition music interventions could become a standard component of sport psychology programs for shooters, as common as imagery or goal-setting, but this will require continued collaboration between scientists and practitioners in the coming years.

## 5 Conclusion

Pre-competition music intervention emerges from this review as a promising, science-backed tool for enhancing performance in competitive shooting, provided it is applied thoughtfully. Music is not magic—it will not compensate for poor technique or insufficient training—but it can give athletes that extra edge by optimizing their internal state before and during competition. For sports like shooting that demand a delicate balance of calmness and concentration, music offers a way to achieve the *optimal zone* of arousal. The mechanisms at play include physiological calming (reduced heart rate, stable autonomic activity, lower stress hormones), psychological tuning (reduced anxiety, improved mood, enhanced focus), and even the priming of motor circuitry through rhythm and imagery. By carefully selecting music with an appropriate tempo, tailoring the choice to the individual's preferences and cultural context, and controlling the listening conditions, athletes and coaches can harness these effects to improve consistency and precision. In practice, a shooter who incorporates soothing, personally empowering music into their pre-shot routine may find they walk to the line feeling more composed, block out distractions more easily, and recover better from the inevitable stresses of competition.

Crucially, this approach aligns with a broader trend in sports psychology: the recognition that *performance is a holistic mind-body phenomenon*. Just as physical training targets strength or endurance, mental preparation techniques like music target the emotional and cognitive readiness of the athlete. The advantage of music is that it is non-invasive, enjoyable, and easy to implement. In the high-pressure world of elite sports, where margins between victory and defeat are razor-thin, such advantages are not trivial. For shooting athletes, even a slight improvement in stability or a small reduction in anxiety can translate into better scores—especially when it comes to that “critical shot” in a final that determines medals. The strategies detailed in this review provide a framework for integrating music into training regimens and pre-match routines in a systematic way. They encourage experimentation and personalization, acknowledging that the *best* music intervention is the one finely attuned to the athlete's own heart and mind.

In conclusion, the intersection of music and competitive shooting performance exemplifies the fruitful merging of art and science. It leverages music's emotive power and physiological impact in a targeted manner to support athletic excellence. While challenges and unknowns remain—such as quantifying individual differences and perfecting the approach—the current evidence and practical insights suggest that music, when used wisely, can help shooters hit their mark more reliably. This review has highlighted how and why music works as well as how to apply it, paving the way for both improved practice on the range and further scholarly inquiry. By continuing to refine this interdisciplinary understanding, sport scientists and coaches can unlock new levels of performance and well-being for athletes, with melodies and rhythms becoming part of the ammunition in the shooter's arsenal for success.

## 6 Limition

The facilitative role of music in enhancing exercise performance is well recognized. Under controlled conditions, appropriately designed music interventions can further optimize athletes' performance outcomes. Previous research, however, has largely focused on the effects of high-volume and fast-tempo music, which are known to elevate physiological and emotional arousal and consequently improve performance in high-intensity or explosive tasks. In contrast, evidence on the use of music strategies with moderate volume and slow tempo remains scarce. Whether such music induces the theoretically proposed effects of calming the mind, stabilizing attention, and supporting performance in low-arousal or precision-based activities has yet to be empirically demonstrated.

In this study, we examined, from a theoretical perspective, the potential performance-enhancing role of moderate-volume and slow-tempo music in calm or steady-state sports. Our findings remain conceptual and highlight the need for systematic experimental validation. Future investigations should specifically address: (1) the effects of moderate-volume, slow-tempo music on sports characterized by low physiological and psychological arousal, such as shooting and archery; (2) the comparative influence of different combinations of musical tempo and volume on performance in these sports; and (3) the potential benefits of such music interventions when applied at different temporal stages—before, during, and after competition—on performance consistency, recovery, and final outcomes.

Furthermore, the present analysis is constrained by a limited disciplinary scope. A more comprehensive framework incorporating insights from medicine, psychology, and biomechanics will be essential to elucidate the mechanisms by which musical parameters modulate physiological states, emotional regulation, and motor performance in calm and precision-demanding sports.
